# Synergistic interactions of cytarabine-adavosertib in leukemic cell lines proliferation and metabolomic endpoints

**DOI:** 10.1016/j.biopha.2023.115352

**Published:** 2023-08-24

**Authors:** Gabriel O. Rodríguez-Vázquez, Adriana O. Diaz-Quiñones, Nataliya Chorna, Iris K. Salgado-Villanueva, Jing Tang, Walter I. Silva Ortiz, Héctor M. Maldonado

**Affiliations:** a Pharmacology Department, Universidad Central del Caribe, School of Medicine, PO Box 60327, Bayamón, PR 00960-6032, USA; b Biochemistry Department, University of Puerto Rico Medical Sciences Campus, PO Box 365067, San Juan, PR 00936-5067, USA; c Department of Biochemistry and Developmental Biology, Faculty of Medicine, University of Helsinki, Haartmaninkatu 8, Helsinki 00290, Finland; d Research Program in Systems Oncology, Faculty of Medicine, University of Helsinki, Haartmaninkatu 8, Helsinki 00290, Finland; e Physiology Department, University of Puerto Rico Medical Sciences Campus, PO Box 365067, San Juan, PR 00936-5067, USA

**Keywords:** Leukemia, WEE1, Synergy, Metabolome, Jurkat, CCRF-CEM

## Abstract

Drug synergy allows reduced dosing, side effects and tolerance. Optimization of drug synergy chemotherapy is fundamental in acute lymphocytic leukemia and other cancers. This study aimed to analyze the pharmacodynamic synergy between the anti-metabolite cytarabine and WEE1 inhibitor adavosertib on acute leukemia cell lines CCRF-CEM and Jurkat. In both cell lines analysis of concentration-inhibition curves of adavosertib-cytarabine combinations and synergy matrixes supported mutually synergistic drug interactions. Overall mean ( ± SD) synergy scores were higher in Jurkat than CCRF-CEM: Jurkat, ZIP 22.51 ± 1.1, Bliss 22.49 ± 1.1, HSA 23.44 ± 1.0, Loewe 14.16 ± 1.2; and, CCRF-CEM, ZIP 9.17 ± 1.9, Bliss 8.13 ± 2.1, HSA 11.48 ± 1.9 and Loewe 4.99 ± 1.8. Jurkat also surpassed CCRF-CEM in high-degree synergistic adavosertib-cytarabine interactions with mean across-models synergy values of ~89.1% ± 2.9 for 63 nM cytarabine-97 nM adavosertib (91.4% inhibition synergy barometer). Combination sensitivity scores scatter plots confirmed combination’s synergy efficacy. This combined approach permitted identification and prioritization of 63 nM cytarabine-97 nM adavosertib for multiple endpoints analysis. This combination did not affect PBMC viability, while exhibiting Jurkat selective synergy. Immunoblots also revealed Jurkat selective synergistically increased γH2AX phosphorylation, while CDC2 phosphorylation effects were attributed to adavosertib’s WEE1 inhibition. In conclusion, the high synergistic efficacy combination of cytarabine (63 nM) and adavosertib (97 nM) was associated with remarkable alterations in metabolites related to the Krebs cycle in Jurkat. The metabolic pathways and processes are related to gluconeogenesis, amino acids, nucleotides, glutathione, electron transport and Warburg effect. All above relate to cell survival, apoptosis, and cancer progression. Our findings could pave the way for novel biomarkers in treatment, diagnosis, and prognosis of leukemia and other cancers.

## 1. Introduction

Leukemia accounts for approximately 2.5% of all new cancer incidence and 3.1% of cancer-related mortality worldwide with 474,519 new cases and 310–330,000 deaths of leukemia reported in 2020, with males population having a mortality rate almost double when compared to women [1,2]. Leukemia ranks 10th in the estimated number of cancer deaths worldwide [1,2]. Of the four main types of leukemias, acute lymphoblastic leukemia (ALL) has the second highest mortality rate of leukemias worldwide with 79,662 deaths (14.24% of total leukemia deaths) reported in 2020 [2]. Globally ALL is the principal contributor to incident cases in individuals between the ages of 0 and 59 years old, and the age-standardized incidence rate is expected to increase for all sub-types of leukemia, with the highest increase (31.1%) predicted for ALL [2]. In the United States leukemia is the ninth highest cancer type to produce new cases in men and tenth in women and the estimated deaths for 2023 have leukemia placed in the sixth position for highest deaths in men and seventh for women [3].

Indeed, acute lymphoblastic leukemia (ALL) is a rapidly progressing, low survival rate cancer mostly affecting children [4-6]. Current therapies for leukemia focus on DNA damaging treatments (DNAdt) such as chemo and radiation therapies. Cytarabine (cytosine arabinoside or ara-C), is widely used in ALL, acute myeloid leukemia (AML), chronic myelogenous leukemia (CML), and non-Hodgkin’s lymphoma [7]. This antimetabolite and nucleoside analog can produce DNA damage, inhibition of both DNA and RNA polymerases, and cell death. Like many DNAdt, cytarabine produces many debilitating side effects, toxicities, and rapid development of resistance. Cytarabine is still used as induction and consolidation phase therapy in ALL and other cancers. Several cytarabine resistance development mechanisms include inactivation by deamination and increase in cell time for DNA repair by overexpression of the G2-M checkpoint kinase Wee-1 (Wee1) [8]. Wee1 inhibitors including adavosertib (MK-1775, AZD-1775) have been shown to overcome chemo-resistance in a variety of cancer models [8]. Adavosertib and Wee1 inhibitors (Zn-C3; Debio 0123; SY4835; and IMP7068) are promising in ALL and other cancers as single and drug combination therapy [4] as revealed by prior and ongoing clinical trials [4,9,10].

Furthermore, Wee-1 is overexpressed in most cancer cells and phosphorylation of its immediate substrate (CDC2; CDK1) will halt cell cycle progression at the G2-M phase [11]. Delay in G2-M phase transition allows more time for DNA repair, allowing the cancer cell to enter mitosis “undamaged”. Inhibition of Wee1 abrogates the extended pause in the G2-M catapulting cancer cells into a mitotic catastrophe and apoptosis. Adavosertib has undergone a series of clinical trials [12], including as single agent treatment in patients with refractory solid tumors [13]. The drug has a proliferation inhibitory effect and induces apoptosis in sarcoma and acute lymphoblastic leukemia cells [14,15]. In combination with other drugs, such as bortezomib, apoptosis was induced in multiple myeloma (MM) cell lines, more efficiently than utilizing isolated drugs [16]. Adavosertib sensitized myeloid leukemia cells to the inhibitory effects of cytarabine (AML, CML and MDS cells) and induction of apoptosis in myeloid cancer cells [17]. In the Jurkat ALL model adavosertib and cytarabine interactions were reported using a limited set of drug combinations and synergy analysis tools [18].

Drug synergy permits enhanced therapeutic efficacy at lower drug concentrations (chemosensitization), concomitant diminished drug(s) toxicity and decreased development of refractoriness-tolerance. Today, expanded models (Loewe, Bliss, highest single agents (HSA), and zero interaction potential (ZIP)) and web-based methods (Compusyn, Combenefit, SynergyFinder Plus, MuSyc, others) provide additional analytical capabilities [19]. We hypothesize and demonstrate that a comprehensive drug synergy analysis in leukemic cells permits selection and prioritization of highly synergistic drug combinations for multiple endpoints assessment. Specifically, our study focuses on cytarabine and adavosertib in the Jurkat and CCRF-CEM ALL model systems. A stepwise approach was applied in the selection and prioritization of drug combinations displaying high degree of synergistic interactions and efficacy to evaluate the effect in highly proliferating non-cancerous T-Cells, low proliferating PBMC’s from a non-cancerous donor, and a series of mechanistic end points of the cell cycle, DNA damage and the Jurkat metabolome. The results shed light on the translation of this drug combination in ALL treatment and provide a framework for synergy-based pharmacometabolomics in cancer.

## 2. Materials and methods

### 2.1. Cell lines, reagents, and culture conditions

Human cell lines Jurkats Clone E6–1 (ATCC TIB-152) and CCRF-CEM (ATCC CCL-119) were obtained from American Type Culture Collection (ATCC) (Virginia, USA) as mycoplasma-free cell lines authenticated by comparing their STR profile with the ATCC Human Cell STR Database. Human cell lines Jurkats Clone E6–1 and CCRF-CEM obtained from ATCC, were maintained in RPMI 1640 medium supplemented with 10% FBS, 1% Antibiotic/antimycotic cocktail, at 37 °C in a 5%CO_2_ atmosphere. PBMC were extracted essentially as previously described [20] using Ficoll gradient centrifugation and cultured in RPMI 1640 medium with 10% heat inactivated FBS, 1% Antibiotic/antimycotic cocktail, at 37 °C in a 5%CO_2_ atmosphere. For experimental usage 6–14 cell passages for Jurkats and CCRF-CEM. T-Cells were isolated using Depletion Dynabeads and activated using Human T-Activator CD3/CD28 Dynabeads and rIL-2 (Invitrogen). Cytarabine was obtained from Sigma-Aldrich (Catalog number: PHR1787) and adavosertib (MK1775) from ChemBlocks Inc. (Catalog number: M15123). Both PBMC’s and T-Cells were extracted from healthy individuals following protocol approval from our Institutional Review Board (IRB).

### 2.2. Concentration-inhibition (dose-response) curves (CIC)

Using Falcon Corning black 384 well plates, single and combined doses of adavosertib and cytarabine were analyzed by Alamar Blue [21, 22] staining using a Victor Wallack plate reader. Cell growth curves at 96hrs determined that 4 × 10^4^/40 μL per well was an acceptable amount to maintain a logarithmic growth of the cells. Concentration-inhibition (dose-response) curves (CIC) were done using 96 low binding plates for preparation of different drug concentrations and adding serial dilutions vertically and horizontally. After 96hrs cells were treated with Alamar Blue incubated for 3hrs and analyzed. Statistical analysis was done using GraphPad Prism, and synergy analysis via web-based freeware Synergyfinder Plus (https://synergyfinderplus.org) [19], Combenefit [23] and Compusyn [24].

### 2.3. Synergy methods

The effects on leukemic Jurkat and CCRF-CEM cell proliferation of cytarabine, adavosertib, and their combinations were analyzed via a series of synergy models (Loewe, Bliss, highest single agents (HSA), and zero interaction potential (ZIP)) using Combenefit and SynergyFinder Plus. CIC data was transformed to data files according to the freeware’s format and further presented in color-coded graphical representations of heatmaps or matrix plots indicating synergy, no effect or antagonism. In the ZIP synergy model synergy scores (sigma) higher than 10 are the most interesting or significant. Across synergy models synergy scores of the most synergistic drug combination concentration ranges were organized in table format. Further pioritization and selection of the synergistic drug combinations for further biomarker analysis was done by CSS versus Synergy scores scatter SS plots, and by using a synergy barometer, where the actual drug combination response can be directly compared with the expectations of non-interaction among multiple synergy models [19]. This identification and selection were also supported using Compusyn-generated isobologram and Fraction affected (Fa)-Combination Index (CI) plots.

### 2.4. Western blots

Jurkat cells were seeded and cultured in 6 well plates and drugs administered at the desired concentrations. After 24 h treatments samples were collected, centrifuged, and washed with 4 mL PBS. Pellets were resuspended in PBS (300 μL), transferred to 1.5 mL conical tubes and mixed with cold acetone (1.2 mL) for storage overnight at −20 °C. Samples were then spun at 12,000 g for 10 min at 4 °C, supernatant discarded, residual acetone extracts dried in a speed vac, and resuspended in DDH2O, phosphate and protease inhibitors. After protein content estimates (Bradford assay) 10 μg protein were loaded per well, ran in 4–20% Mini-protean^®^ precast gels (Bio-Rad) and transferred to PVDF membranes overnight. Membranes were washed with PBS/0.4% Tween20 (5 min twice), stained with Indian ink (1 hr 30 min), washed with 1X TBS and dried. An image from each stained membrane was obtained for total protein quantification, and subsequent analysis done with Molecular Imager^®^ VersaDoc^™^ MP Imaging Systems (Bio-Rad). After drying, rehydration (in methanol), and blocking (5% BSA, 0.5% Tween20, 1X TBS 20 mL) membranes were incubated with primary (Cell Signaling Technology^®^) and secondary antibodies (Sigma^®^) diluted in blocking solution: pCDC2 (Y15) (1:5,000) (Cell Signaling Technology, Catalog # 4539 S), H2Ax (S139) (1:25000) (Cell Signaling Technology, Catalog # 9718 S) and Goat anti-rabbit (2ry) antibody (1:100,000) (Sigma Catalog # SAB3700878, Lot # R135256). Chemiluminescent signal detection was done with SuperSignal^™^ West Femto Maximum Sensitivity Substrate (West Thermo Scientific^™^), membranes processing and analysis with ChemiDOC XRS+ System^™^ Imaging System (Bio-Rad) and analysis with Image Lab Software (Bio-Rad) following manufacturer’s instructions.

### 2.5. Metabolomic extraction

After treating leukemic Jurkat cells for 48 h with cytarabine (63 nM), adavosertib (97 nM) and their combinations samples (~10^7^ cells per sample) were homogenized in methanol (1 mL, 99%), homogenates shaken (15 min) and centrifuged at 13,000 rpm (10 min) (all at 4°C). Supernatants were evaporated, metabolites derivatized by methoxyamination in a solution of methoxyamine hydrochloride (Sigma-Aldrich) in pyridine (Sigma-Aldrich) and incubated at 37 °C for 2 h. Trimethylsilylation subsequently done adding of N-methyl-N-trimethylsilyl-trifluoroacetamide (MSTFA+1% TMCS) (Sigma-Aldrich) (50 μL). Samples were incubated (65 °C, 1 hr), centrifuged (3000 rpm, 10 min at RT), and supernatants transferred to glass vials. Samples (20 μL) were added to glass vials with inserts and 1 mM 2-Fluobiphenyl (Sigma-Aldrich) added as internal standard, and processed by gas chromatography-mass spectrometry GC/MS-QP2010 (Shimadzu, Inc.) [25-27].

### 2.6. Data processing and bioinformatics

Raw chromatography data were obtained and processed in GCMS Solution Post run Analysis software (Shimadzu Scientific Instruments, Inc., Columbia, MD) equipped with NIST14/2014/EPA/NIH database. Peak integrations of metabolites and extensive mass spectral library searches of major peaks yielded a final data set of 22 metabolic features selected for metabolomics analysis. Reproducibility of metabolite recovery, performance of sample extraction, derivatization, and instrumentation were validated by utilization of several blank samples for system suitability, extraction processing, and derivatization processing. For reproducibility of metabolic features, a pooled composite sample was prepared from each experimental sample, aliquoted and processed with experimental samples as quality control (QC) (n = 5). Systematic bias mitigation was done via randomization of sample analysis order. Blanks and QC samples were spaced evenly among injections to monitor instrument stability. Identified metabolites were transferred to a data matrix alongside retention time, peak area, and reference ions per metabolite. Quantitative analysis of metabolic feature’s concentrations per sample done by calculation of a response factor using the internal standard. A table containing metabolite concentrations per sample was analyzed via MetaboAnalyst 5.0 [28]. Samples were normalized by sample weight and range scaling. Data processed and statistically analyzed using multivariate, and univariate analyses. Samples were normalized by sample weight and range scaling. Multivariate analyses consisted of principal component analysis (PCA) for variance between samples and partial least squares-discriminant analysis (PLS-DA) to identify groups separation. Quality and reliability were assessed by cross-validation using parameters R2 and Q2 (R2 measures degree of goodness of fit of the data; Q2 measures quality assessment). PLS-DA significance was assessed by Permutation Test, with ≤ 0.05 considered statistically significant. Univariate analyses between sample treated with cytarabine, adavosertib, the drug combination and control groups were assessed by one-way ANOVA with Fisher’s LSD post hoc test with p ≤ 0.05 considered statistically significant. PLS-DA, and Heatmap plots were generated via MetaboAnalyst [28]. To gain insights into the underlying biological processes and metabolic functions affected by cytarabine, adavosertib, and their combinations, we conducted the metabolite set enrichment analysis (MSEA) using Metaboanalyst 5 [28].

## 3. Results

### 3.1. Concentration-inhibition curves of adavosertib and cytarabine in CCRF-CEM and Jurkat cells

**Fig. 1.**
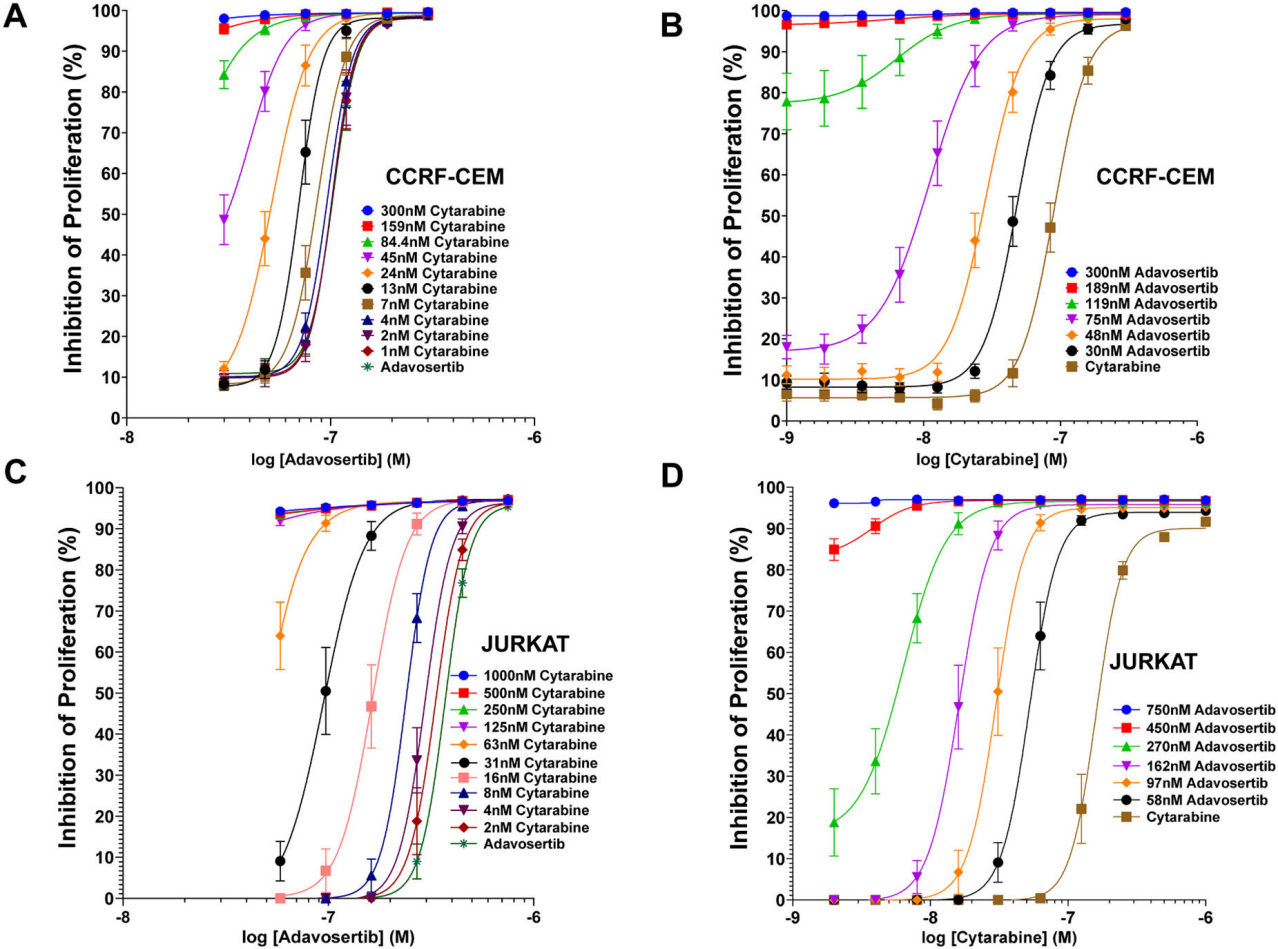
Concentration Inhibition Curves (CIC) of Adavosertib, Cytarabine and their Combinations in CCRF-CEM and Jurkat Leukemic Cell Lines. Panel A, CCRF-CEM CIC curves of adavosertib alone or combined with different fixed concentrations of cytarabine. Panel B, CCRF-CEM CIC curves of adavosertib alone or combined with different fixed concentrations of cytarabine. Panel C, Jurkat CIC curves of adavosertib alone or combined with different fixed concentrations of cytarabine. Panel D, Jurkat CIC curves of cytarabine alone or combined with different fixed concentrations of adavosertib. The second drug concentrations are included in each panel inserts with the corresponding color-coded symbols. Each data point represents the mean inhibition value with the standard error mean (SEM) (n = 8).

Concentration-inhibition (dose-response) curves (CIC) determined the relative potency and efficacy of adavosertib, cytarabine and their combinations inhibiting proliferation of ALL CCRF-CEM (Figs. 1A and 1B) and Jurkat cells (Figs. 1C and 1D). The potency of adavosertib was higher in CCRF-CEM (IC50 ~103 nM ± 5 nM) (Fig. 1A, Table 1) than Jurkat (IC50 ~ 370.4 nM ± 13 nM) (Fig. 1C, Table 1). The potency of cytarabine was also higher in CCRF-CEM (IC50 ~90 nM ± 5 nM) (Fig. 1B, Table 1) than Jurkat (IC50 ~159.7 nM ± 8 nM) (Fig. 1D, Table 1). In both leukemic cell lines, the increase in the apparent potency and efficacy (parallel left shifts and upward trends) of the CIC for adavosertib in the presence of increasing concentrations of cytarabine, and the CIC curves of cytarabine in the presence of increasing concentrations of adavosertib are strongly suggestive of synergy. The magnitude of parallel left shifts in the CIC of CCRF-CEM yields a maximum 2.02-fold decrease in the IC50’ of adavosertib in the presence of 24 nM cytarabine (Fig. 1A, Tables 1) and 8.18-fold decrease in the IC50’ for cytarabine in presence of 75 nM adavosertib (Fig. 1B, Table 1). In Jurkat there is a maximum 3.91-fold decrease in the IC50’ of adavosertib in the presence of 31 nM cytarabine (Fig 1C, Tables 1) and 9.86-fold decrease in the IC50’ for cytarabine in presence of 162 nM adavosertib. All together the CIC potentiation data is strongly supportive of the drugs’ mutual synergistic interaction with the largest magnitude changes obtained in Jurkat cells (Table 1).

### 3.2. Synergy Analysis of Adavosertib and Cytarabine Combinations in CCRF-CEM and Jurkat Cells

**Fig. 2.**
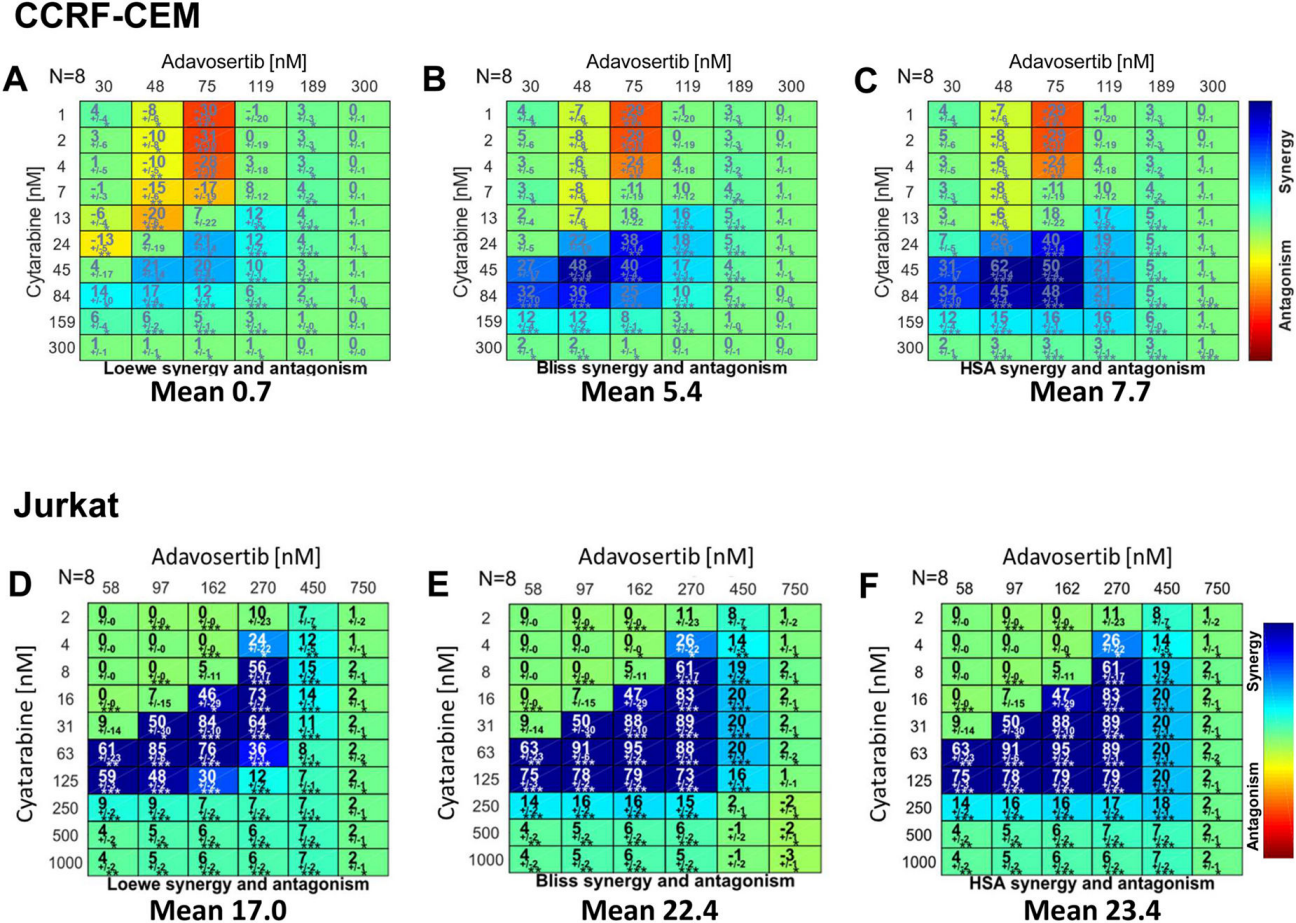
Combenefit Synergy Matrixes for Adavosertib-Cytarabine in the CCRF-CEM and Jurkat Leukemic Cell Lines. The figure illustrates the Combenefit-generated matrixes of the drug combinations demonstrating with shades of blue showing the levels of synergy corresponding to different models (color scales to the right of all panels) with the overall mean synergy score indicated at the bottom of each matrix. Panels A-C correspond to CCRF-CEM cells: (A) Loewe additivity, (B) Bliss independence, and (C) Highest Single Agent Model (HSA). Panels D-F correspond to Jurkat cells: (D) Loewe additivity, (E) Bliss independence, and (F) Highest Single Agent Model (HSA). Values indicate the mean synergy score with the standard error mean (SEM) (n = 8), the matrix of drug combinations with degree of significance (*) (t-test) calculated by the program as a synergy score (n = 8). Statistical significances: * , p < 0.05; * *, p < 0.01; * ** , p < 0.001.

**Fig. 3.**
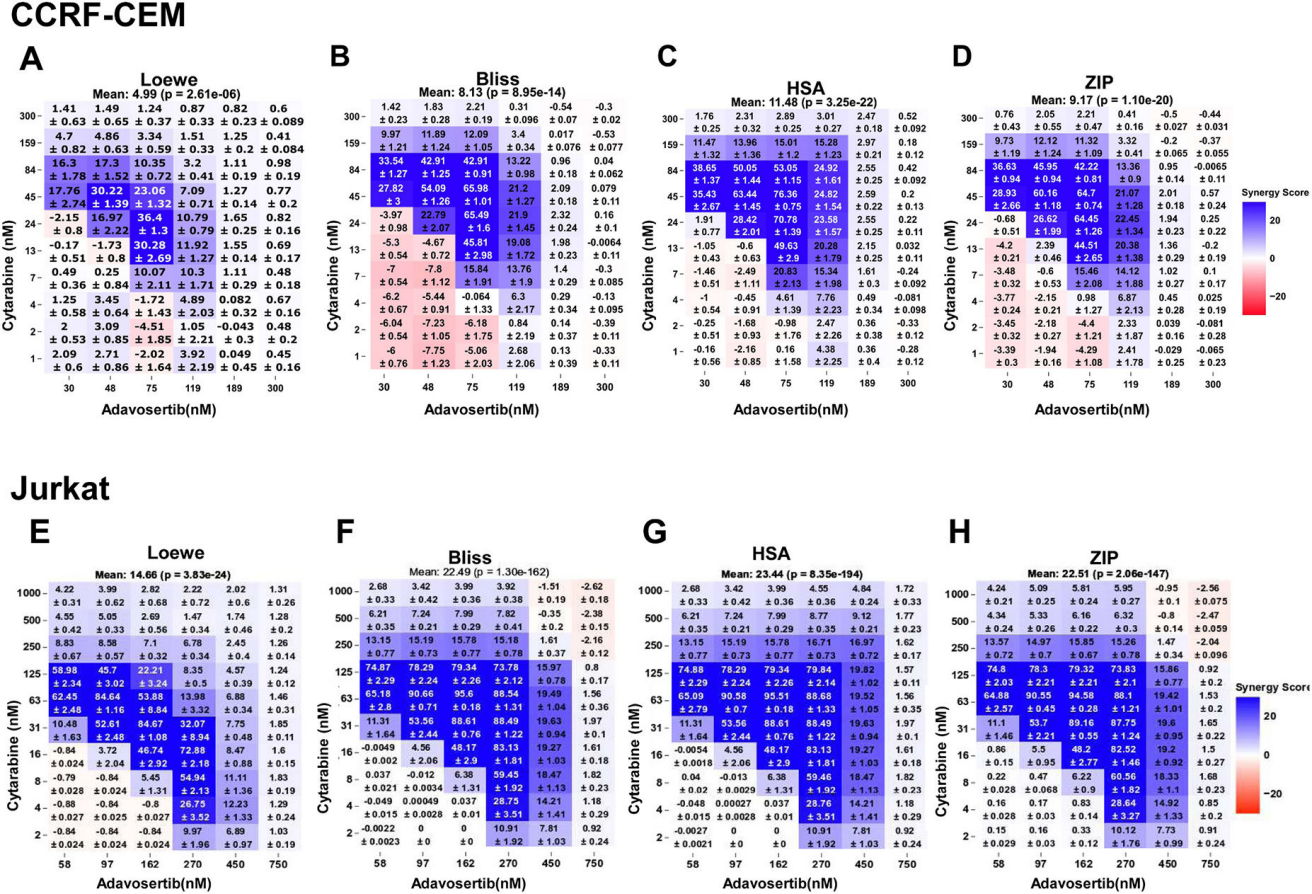
SynergyFinder Plus-generated Synergy Matrixes for Adavosertib-Cytarabine in the CCRF-CEM and Jurkat Leukemic Cell Lines. The figure illustrates the SynergyFinder Plus-generated matrixes of the drug combinations demonstrating with shades of blue showing the levels of synergy corresponding to different models (color scales to the right of all panels) with the overall mean synergy score indicated at the top of each matrix. Panels A-D correspond to CCRF-CEM cells: (A) Loewe additivity, (B) Bliss independence, (C) Highest Single Agent Model (HSA), and (D) Zero Interaction Potential (ZIP). Panels E-H correspond to Jurkat cells: (E) Loewe additivity, (F) Bliss independence, and (G) Highest Single Agent Model (HSA), and (H) Zero Interaction Potential (ZIP). Values indicate the mean synergy score with the standard error mean (SEM) (n = 8), the matrix of drug combinations with degree of significance (*) (t-test) calculated by the program as a synergy score (n = 8). Statistical significances: * , p < 0.05; * *, p < 0.01; * ** , p < 0.001.

To further establish in both leukemic cell lines the synergistic nature of this drug pair their CIC data sets were analyzed via Combenefit (Fig. 2) which permits analysis using Loewe, Bliss and HSA models, and SynergyFinder Plus (Fig. 3) that adds the ZIP synergy model (a Loewe-Bliss chimera) [29,30]. In CCRF-CEM and Jurkat, both synergy analytical tools yield synergy matrixes with visually discernible areas of high degrees of synergistic interactions within defined concentration ranges (Figs. 2 and 3). In CCRF-CEM the respective overall synergy scores (Combenefit, SynergyFinder Plus) per model were Loewe (0.7, 4.99), Bliss (5.4, 8.13), HSA (7.7, 11.48) and ZIP (9.17) (Figs. 2 and 3). While in Jurkat, the respective overall synergy scores (Combenefit, SynergyFinder Plus) per model were higher: Loewe (17.03, 14.67), Bliss (22.25, 22.49), HSA (23.73, 23.44) and ZIP (22.51) (Figs. 2 and 3). Hence, irrespective of the freeware or synergy model, the extent and overall synergy values in Jurkat also surpassed those determined for CCRF-CEM (Figs. 2 and 3. Analysis in both leukemic cell lines of the adavosertib-cytarabine concentration pairs displaying the highest degrees of synergy also unveiled higher average synergy values for Jurkat over CCRF-CEM with all synergy models and analytical tools (Table 2).

### 3.3. Identification and prioritization of synergistic adavosertib-cytarabine drug combinations

Given the higher degree of synergistic interactions determined in Jurkat, this leukemic cell line was subsequently used for the identification, selection and prioritization of the adavosertib-cytarabine concentration(s) pair to be used for diverse cellular, molecular and metabolomic endpoint analysis. Table 2 summarizes the top overlapping high synergy adavosertib-cytarabine combinations per synergy model derived from the corresponding synergy matrixes (Figs. 2 and 3). In Jurkat, across model highest mean synergy values for adavosertib-cytarabine concentration pairs were of 89.0% (Combenefit) and 89.1% (SynergyFinder Plus) for 97 nM adavosertib-63 nM cytarabine (Table 2). In contrast, CCRF-CEM yielded lower across model highest mean synergy values of 43.7% for 48 nM adavosertib-45 nM cytarabine (Combenefit) and 59.3% for 75 nM adavosertib-24 nM cytarabine (SynergyFinder Plus) (Table 2).

**Fig. 4.**
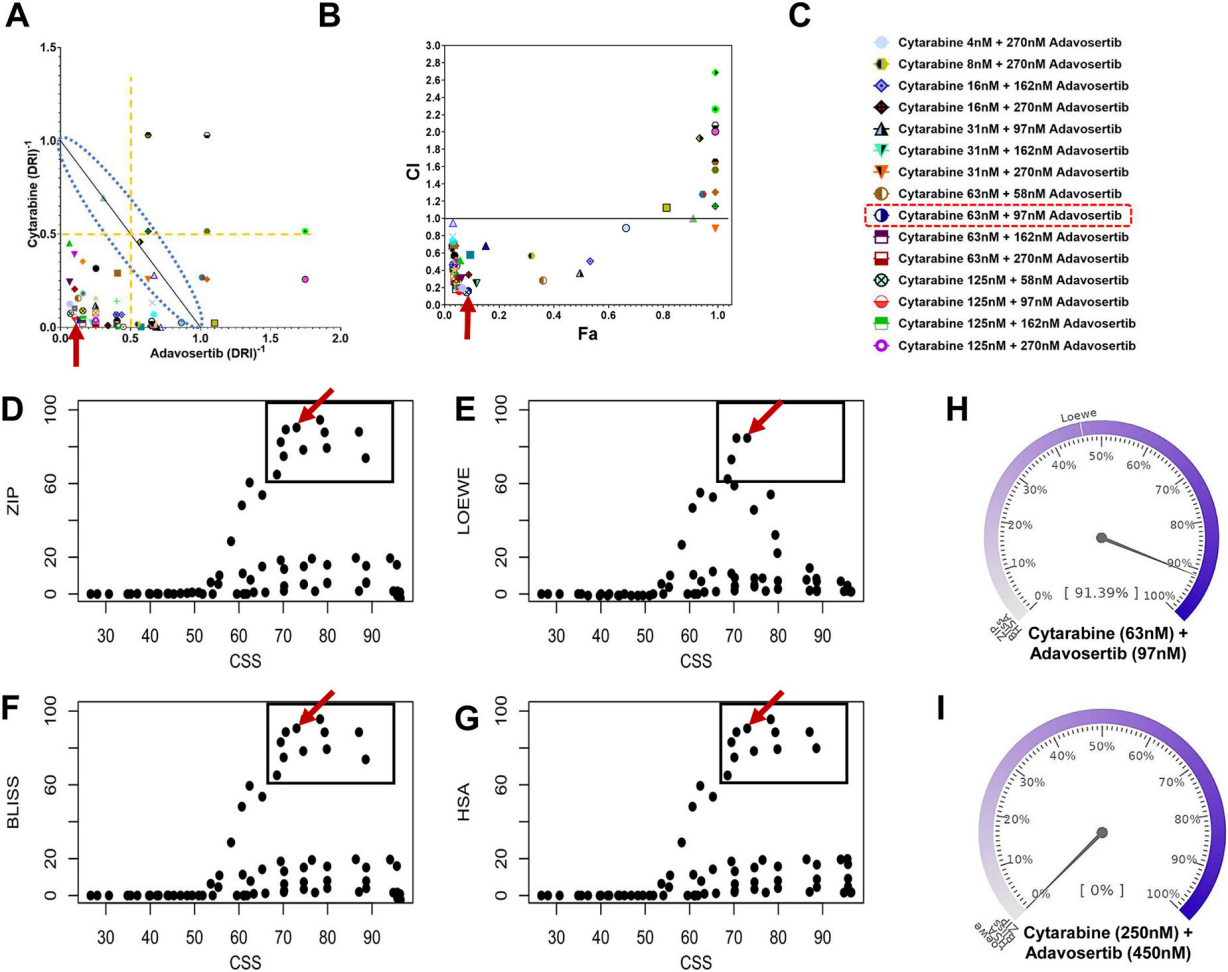
Isobologram, Fa-CI Plot, Scatter SS Plots and Barometers for Adavosertib-Cytarabine in the Jurkat Leukemic Cell Line. Compusyn generated isobologram (Panel A) and Fa-combination index (CI) (Panel B) plots. In both plots the highest synergy scores are located within the lower quadrant well below the lines of additivity (dashed blue lines envelope in Panel A). The red arrows identifies our combination of interest and highest degree of synergistic interaction cytarabine (63 nM) and adavosertib (97 nM). Panel C includes figures symbols corresponding to the top 15 most synergistic combinations (see Table 2). Panels D-G are the corresponding scatter SS Plots (CSS versus Synergy Score) for the different synergy models of Jurkat cells. CSS indicates the efficacy of a drug combination, whereas the synergy score for each model indicates the degree of interactions. The upper right quadrant of the plots depicts cytarabine-adavosertib combinations exhibiting higher CSS and higher synergy scores. The plots further support the prioritization of the 63 nM cytarabine with 97 nM adavosertib drug combination pair for further endpoint analysis (red arrow). Panel H illustrates the synergy barometer for the 63 nM cytarabine-97 nM adavosertib combination exhibiting a 91% inhibition, and for comparison Panel I barometer shows 0% inhibition for 15 nM cytarabine with 58 nM adavosertib (AZD-1775). In the barometer the needle indicates the overall percent inhibition and lines indicate the relative levels per synergy model.

In Jurkat, the high degree of synergy of 97 nM adavosertib-63 nM cytarabine was also seen in the normalized isobologram and Fa versus CI plot (Fig. 4A-C, red arrows). In both plots the high degree of synergy drug combinations are found within the lower left quadrants, well below the lines defining additivity. Furthermore, combination sensitivity scores (CSS) versus synergy scores scatter plots (SS plots) for all models confirmed the high synergy efficacy and high degree of synergistic interaction of the 97 nM adavosertib-63 nM cytarabine combination (Fig. 4D-G). In addition, using a synergy barometer, the actual drug combination response was directly compared with expectations of non-interaction among all synergy models [19]. Fig. 4H displays the synergy barometer for 97 nM adavosertib-63 nM cytarabine which reaches 91.39% inhibition (see pointer readout on the barometer). The drug combination responses display high-degree synergistic interaction as expected responses of HSA, Loewe, Bliss, and ZIP models are much smaller, shown as the marks on the barometer [19] (Fig. 4I is a control non-synergistic drug combination). Therefore, the 97 nM adavosertib-63 nM cytarabine combination was selected and prioritized for subsequent analysis on cellular, molecular and metabolomic endpoints.

### 3.4. Synergistic cytarabine-adavosertib combination effects on PBMC and activated T-Cells

**Fig. 5.**
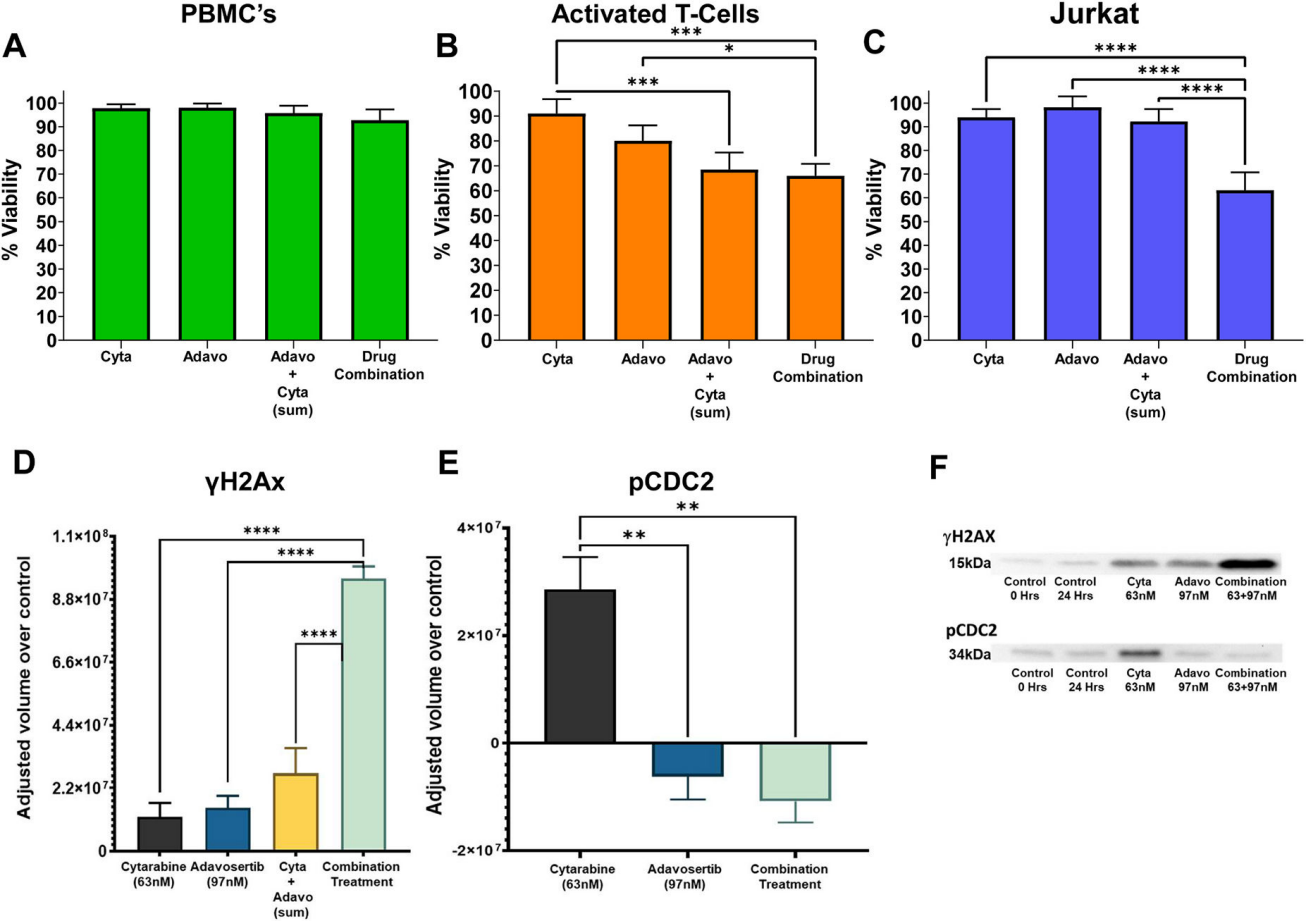
Effect of Cytarabine and Adavosertib Alone and in Combination on Cell Viability of Non-proliferating PBMC’s, Activated T-Cell’s and Leukemic Jurkat Cells; and, two Mechanistic End Points Related to the Cell Cycle and DNA Damage. The effects of the highly synergistic concentrations of cytarabine (63 nM), adavosertib (97 nM) as single drug treatment and in combination are shown for normal PBMC (A), activated T-cells (B), and Jurkat (C) at 48Hrs. The data reveal a lack of effect of the treatments on PBMC viability, a decreased viability produced by either drug and their combination on activated T cells and Jurkat. Interestingly, only the effect of the drug combination was significantly higher than the effects of either drug or the sum of their individual actions, akin a Jurkat–selective synergistic effect. Experiments were conducted using Trypan blue viability staining to compare the viability percentage. Statistical analysis was conducted using Tukey’s test with the standard error mean (SEM) (n = 4). Statistical significances: * , p < 0.05; * *, p < 0.01; * ** , p < 0.001; * ** *, p < 0.0001. Panel D-F and Fig. S1 represents the quantitative densitometric analysis of immunoblot data normalized over the 24hrs control. Panel D shows an increase in γH2AX phosphorylation elicited by the high synergistic combination and the single doses of cytarabine (63 nM) and adavosertib (97 nM). Interestingly, as in the viability assays on Jurkat (Panel C), only the effect of the drug combination was significantly higher than the effects of either drug or the sum of their individual actions akin a Jurkat–selective synergistic effect. Panel F display immunoblots of the phosphorylation of the G2/M cell cycle progression marker CDC2 (34 kDa) and the DNA damage marker γH2AX after 0 and 24hrs exposure to the drugs alone or in combination. Panel E is the quantitative densitometric analysis using India ink revealing a significant increase in phospho-CDC2 elicited by cytarabine consistent with its ability to induce DNA damage and delay cell cycle progression. In turn, adavosertib, consistent with its WEE1 kinase inhibition, significantly decreased CDC2 phosphorylation when combined with cytarabine. Statistical analysis was conducted using Tukey’s test with the standard error mean (SEM) (n = 4). Statistical significances: * , p < 0.05; * *, p < 0.01; * ** , p < 0.001; * ** *, p < 0.0001.

The high degree of synergy interaction and efficacy 97 nM adavosertib-63 nM cytarabine combination was further evaluated on the viability of Jurkat, PBMC and activated T-cells. Non-proliferating PBMC were not affected by any drug treatment. In Jurkat and activated T-cells the combination significantly diminished cell viability (Fig. 5A-C). In activated T-cells the combination and sum of the individual actions of both drugs were significantly higher than 63 nM cytarabine. The drug combination was not significantly different from the sum of both drugs as predicated by the additivity principle. Most importantly, in Jurkat the combination treatment was significantly higher than the individual drugs (HSA principle) and the sum of their effects (additivity principle). The results suggest that this type of treatment is not ALL specific and may target other proliferating cells. The selective synergistic action on Jurkat cells over non-cancerous cells delineates another advantage of synergistic drug interactions, cancer-cell selective synergy.

### 3.5. Effects of synergistic cytarabine-adavosertib combination on mechanistic end points related to the cell cycle and DNA damage in Jurkat

Diverse mechanisms of anticancer drugs affect cell cycle progression and induction of DNA damage. These combined effects can lead to mitotic catastrophe of cancer cells. Hence, the impact of cytarabine, adavosertib and their high-synergy combination was determined on the phosphorylation of DNA damage biomarker γH2AX (Figs. 5D, 5F) and phosphorylation of CDC2 a cell cycle progression biomarker (Figs. 5E, 5F). Adavosertib (97 nM) and Cytarabine (63 nM) produce a slight but not significant increase in γH2AX phosphorylation, while the drug combination produces a highly significant increase of its phosphorylation (Fig. 5D). The combination effect was significantly higher when compared to the control group, each individual drug, and the sum of the individual effects (Fig. 5D), hence supporting their synergistic interaction. Fig. 5E shows a significant increase in pCDC2 after treatment with cytarabine when compared to all other groups. In contrast, adavosertib alone had no effect in the phosphorylation of CDC2 (Fig. 5E) when compared to the control, while producing a significant decrease when compared to cytarabine alone and an even higher decrease in the combination treatment. The drug combination decreased pCDC2 is attributed and expected due to the inhibition of WEE1 kinase by adavosertib [31].

### 3.6. Assessment of Synergistically Effective Cytarabine-Adavosertib Combination on Jurkat Metabolomic Endpoints

**Fig. 6.**
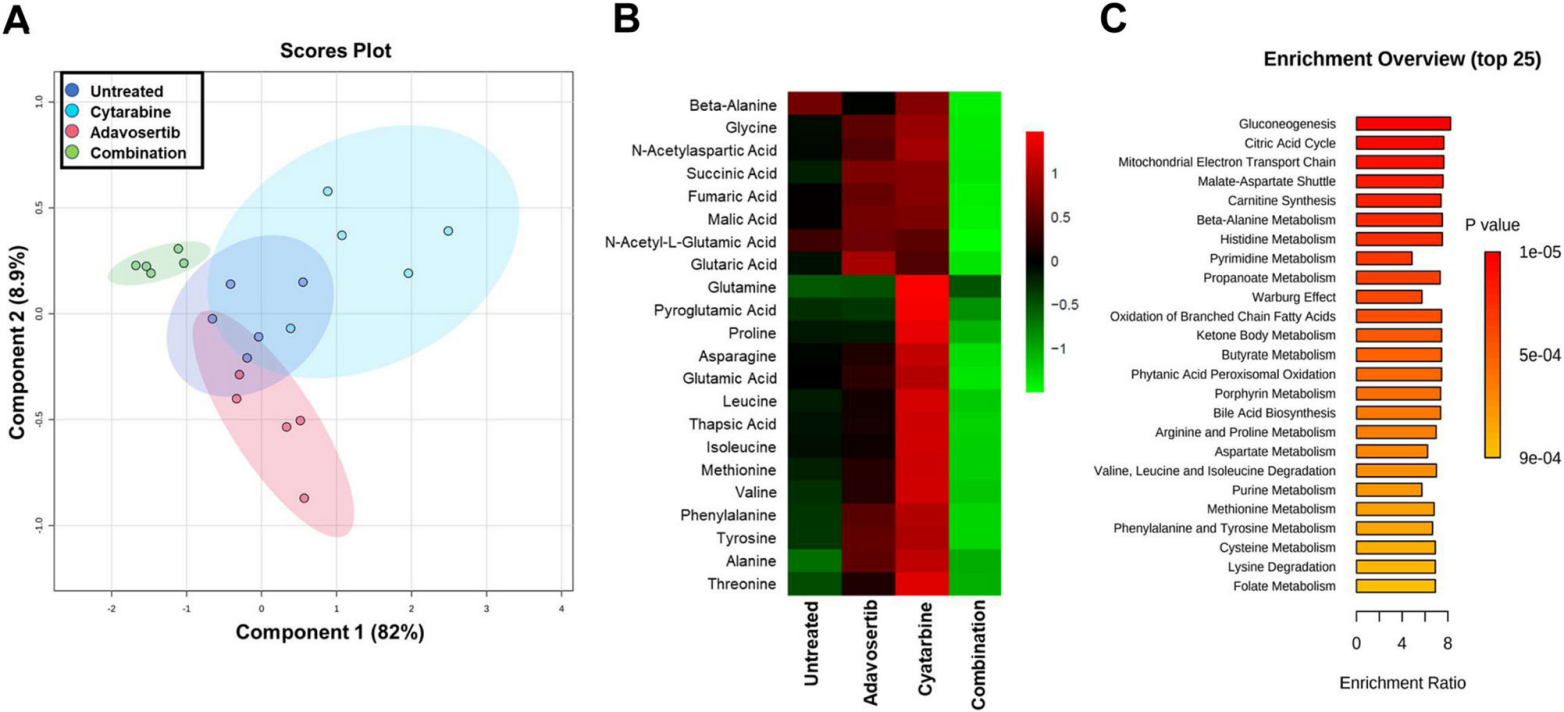
Effect of Cytarabine and Adavosertib Combination on the Jurkat Metabolome. Panel A is the partial least squares-discriminant analysis (PLS-DA) score plot showing the discriminating characteristics of the Jurkat Cell metabolomic endpoints, with a clear clustering of the data points of the different drug treatments (63 nM cytarabine, 97 nM adavosertib and their combination) and the untreated sample control after 48Hrs (see the color codes in the box inside the figure). Consideration of component 1 (82% variance) and to a lesser extent Component 2 (8.9% variance) permitted separation of the synergistic drug combination treatment from the control group and individual drug treatments that showed overlapping segments. Each group consisted of five samples. Panel B reveals the heatmaps for 22 metabolites being differentially affected after 48hrs of treatment with cytarabine (63 nM), adavosertib (97 nM) and their combination versus a control untreated group. Panel C lists the enrichment overview of the top 25 pathways affected by the combination treatment when compared against the untreated control sample (n = 5).

**Fig. 7.**
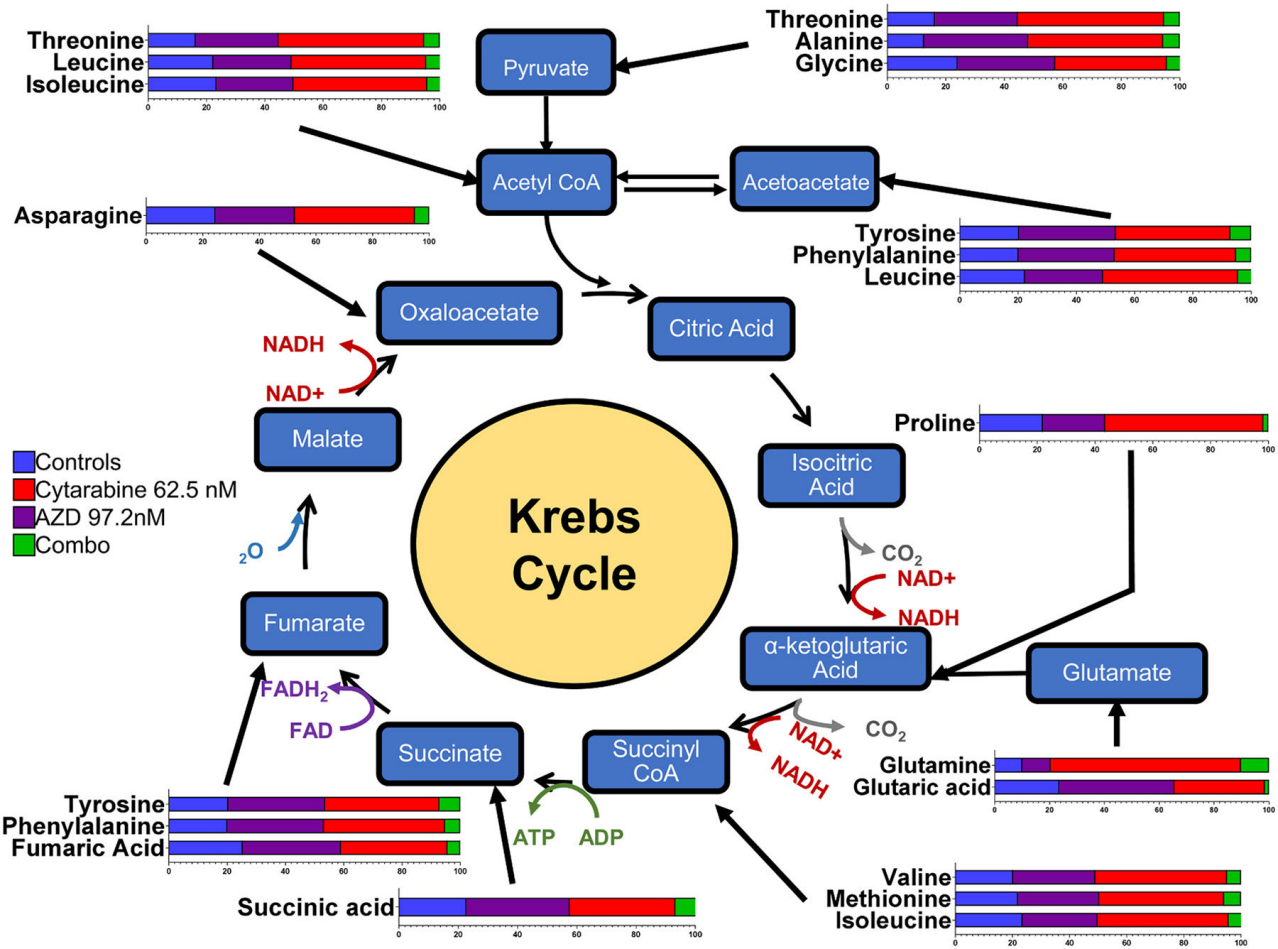
Relationship of the Jurkat Metabolites to the Krebs Cycle. The figure illustrates the relative changes (horizontal bar graphs) in the concentrations of each metabolite (amino acid) and their relationship to the Krebs cycle. The relative changes of each metabolite are further included in supplementary Fig. S2, where statistical analysis was conducted using Tukey’s test with the standard error mean (SEM) (n = 5). Statistical significances: * , p < 0.05; * *, p < 0.01; * ** , p < 0.001; * ** *, p < 0.0001.

After treating Jurkat cells for 48 h with adavosertib (97 nM), cytarabine (63 nM) and their combination samples were analyzed via Gas Chromatography/Mass Spectrometry using NIST14/2014/EPA/NIH database (see Methods). Further analysis with MetaboAnalyst 5.0 [28] detailed components of metabolic pathways affected. A partial least squares-discriminant analysis (PLS-DA) (Fig. 6A) identified the significant separation between groups based on cross-validation and permutation test. There is a clear separation of the combination treatment compared to cytarabine, adavosertib, and control group (the latter three showing partial overlap) (similar patterns were also obtained by analysis of the data via sPLS-DA and PCA methods). In Fig. 6B, the metabolomic heatmap shows the main metabolites (22) affected by the adavosertib-cytarabine drug combination which are amino acids intimately linked to the Krebs cycle (Fig. 7 and Table 3). Despite the apparent increases of these metabolites by the individual drugs, when compared to the control group only ten (10) of the 22 metabolites were significantly increased by cytarabine (63 nM) (Fig. 6B, Fig. S2). The prevailing most consistent and significant changes were the inhibition of the 22 metabolites elicited by adavosertib-cytarabine compared to the control group and individual drugs (Fig. 6B, Fig. 7 and Fig. S2). Analysis of the metabolites whose levels were statistically significantly reduced by the cytarabine-adavoserib combination (Fig. S2 and Fig. 6C) when compared to the control group revealed that the synergistic drug combination elicited statistically significant decreases when compared to the control group in thirteen (13) (asparagine, b-alanine, glycine, valine, phenylalanine, glutaric acid, isoleucine, succinic acid, fumaric acid, malic acid. N-acetyl-aspartic acid, N-acetyl-l-glutamic acid and thapsic acid) of the twenty-two (22) metabolites (Fig S2). Nine (9) of the latter metabolites (asparagine, glycine, valine, phenylalanine, isoleucine, succinic acid, fumaric acid, N-acetyl-aspartic acid and N-acetyl-glutamic acid) have been shown to promote tumor growth and/or metastasis in various types of cancer and leukemia [32-45]. While dual roles have been reported for isoleucine and succinic acid [46-48]. And five (5) of the thirteen (13) metabolites are glucogenic amino acids (asparagine, b-alanine, glycine, valine and phenylalanine) [49] an observation consistent with gluconeogenesis being one the three main enriched processes (Fig. 6C) affected by the drug combination. Further analysis links these metabolites to 44 enriched pathways (top 25 shown in Fig. 6C and Table 3) where the combination treatment had a greater effect on 43 of the top 44 pathways when compared to the single doses. The metabolite changes are also linked to multiple cellular processes as shown in Table 3. The pathways and processes have proven relevance to cancer onset and progression. The analysis presented provides a pharmacometabolomic framework of molecular endpoints to analyze synergistic drug interactions.

The metabolomic findings are a primer for the cytarabine-adavosertib synergistic combination in the ALL model Jurkat, yet it must be noted that our study on the synergistic interactions of cytarabine-adavosertib in leukemic cell lines proliferation and metabolomic endpoints has limitations. The relatively small number of metabolites identified using a GC/MS-based metabolomics approach compared to the broader coverage achievable with LC/MS/MS methods limits the broad exploration of the complete metabolic pathways related to the synergy mechanism. By incorporating LC/MS/MS techniques in future research, this limitation can be surmounted to gain a more comprehensive understanding of the synergy mechanism within the complete metabolic pathway. This will enable us to enhance our insights into the broader metabolic landscape associated with the observed synergistic effects and provide a more thorough characterization of the underlying metabolic alterations.

## 4. Discussion

ALL affects T-Cells and B-Cells demanding aggressive therapeutic treatment [50]. ALL normally affects children but is more deadly in adults over age 60. In contrast, CLL exhibits a slower progression, providing an expanded therapeutic window [51]. Our study expanded analysis of synergism between adavosertib and cytarabine in the CCRF-CEM and Jurkat ALL model systems. An ex vivo study reported adavosertib sensitizes primary AML, MDS, and CML specimens to cytarabine and potentiates their antileukemic activity in myeloid cells [17]. The latter study used cytarabine-advosertib combinations on 6–8 cell lines of AML, CML, ALL (Jurkat cells included) and ex vivo cell cultures. Sensitization studies of cytarabine-adavosertib on ALL cell models determined the IC50 in Jurkat for cytarabine at 58.4 nM and 284.9 nM for adavosertib (72hrs time frame) [18]. In the following ALL cell lines the IC50 for adavosertib were 196.7 nM (HPB-ALL), 261.3 (CCRF-CEM) and 336.4 nM (Jurkat) (Hu et al., 2021). Another study reported IC50s ranging from 300 to 600 nM for adavosertib and cytarabine 75–150 nM [17]. Our IC50 values in Jurkat for both drugs are within the latter ranges being 159.7 nM cytarabine and 370.4 nM adavosertib at 96 h. CCRF-CEM displayed an IC50 of 90 nM for cytarabine and 103 nM for adavosertib. Pharmacodynamic analysis of CCRF-CEM and Jurkat drug combinations CIC (Fig. 1) demonstrated their mutually synergistic interactions as revealed by the increased apparent potencies and efficacies of adavosertib in the presence of cytarabine, and vice versa. The magnitude of the shifts in CIC elicited by the combinations was greater in Jurkat, while adavosertib proved to be most effective chemosensitizing agent in both leukemic cell lines. The differences in adavosertib and cytarabine potencies and degrees of synergistic interactions between CCRF-CEM (T-cell acute lymphoblastic/lymphoma) and Jurkat (T-cell acute lymphoblastic) cells may relate to their origin or relative expression of WEE1 since Jurkat expresses higher WEE1 mRNA than a series leukemic cell lines including CCRF-CEM [52]. Furthermore, decreased expression of WEE1 targets CDK1 (CDC2) and CDK2 abolished the combinatorial chemosensitizing effect of adavosertib (AZD1775) in Jurkat [52,53]. Hence, the combinatorial synergistic efficacy of cytarabine-adavosertib in Jurkat and CCRF-CEM may also be due to the relative expression or activation states of the WEE1 target proteins CDC2 and CDK1 [53]. Our findings are also be consistent with the conclusion that the role of WEE1 in cells with accumulated DNA damage extends beyond regulation of CDK1 and the G2/M checkpoint [53] such as the recently established role of CDK2 in G1/S arrest [11,54].

The adavosertib-cytarabine synergistic interactions was further established using two complementary synergy analytical approaches and frameworks [19]. Previous adavosertib-cytarabine synergy studies in Jurkat used the Bliss synergy model on MacSynergy II [18] and a Loewe based model on CalcuSyn [17]. This prior analysis yielded a single synergistic bar graph [18] and a CI estimates [17] for limited drug combinations. Our study on Jurkat yielded respective average synergy scores per model with Combenefit and SynergyFinder Plus of Loewe (17.03, 14.67), Bliss (22.25, 22.49), HSA (23.73, 23.44) and ZIP (22.51) (Figs. 2 and 3, Table 2). While in CCRF-CEM lower overall mean synergy values were observed. Across models highest mean scores, isobologram, Fa-CI plot (CompuSyn), SS scatter plots, and synergy barometer permitted identification, selection and prioritization of the 97–63 nM cytarabine drug combination. These individual drug concentrations constitute values eliciting ~25% inhibition each or corresponding to ~IC25. As shown, this drug concentration pair displays high degree of synergistic interactions and synergy efficacy, hence it was selected and prioritized for evaluation of the effects on endpoints related to white blood cells proliferation, cell cycle, and Jurkat metabolome.

Our findings demonstrate that the drugs alone and in combination did not affect non-proliferating PBMC but decreased the viability of activated T cells as well as leukemic Jurkat cells (Fig. 5). Hence, the drugs can potentially impact proliferating cells in gastrointestinal mucosa, other mucous membranes, and hematopoietic cells. This outcome resembles a Phase I Study in refractory solid tumor patients where adavosertib’s effects (described as manageable) were seen on hematologic cells and the gastrointestinal tract [13,55]. As other chemotherapies, inhibiting proliferating activated T Cells compromises a normal person and patient’s immune response. Most importantly, synergism was further supported in this Jurkat endpoint as the effect of the drug combination was significantly higher than either drug’s effect (HSA principle) and the sum of their effects (Loewe additivity principle) (Fig. 5), akin a leukemic Jurkat cell–selective synergistic effect. The selective synergistic action on Jurkat cells over non-cancerous cells delineates another advantage of synergistic drug interactions, cancer-cell selective synergy.

It must be noted that although the combination was more effective, it significantly decreased the cell viability of activated T-cells (Fig. 5B). This observation parallels findings with most chemotherapeutic agents on normally replicating cells posing a challenge for determining in humans the most effective and less toxic drug combination to take advantage of their synergistic efficacy. This challenge may be addressed by a priori clinical determination of a patients’ WEE1 expression in PBMC’s and primary leukemic cells since its levels may vary between patients [52]. This approach can be coupled to a clinical in vitro adavosertib-cytarabine combination screening with selected prioritized synergistic drug concentration pairs on isolated patient’s PBMC and primary leukemic cells. Similarly, we must underscore that these are preclinical, in vitro studies which not always may predict complex human conditions in vivo. Hence, the value of our findings can be expanded to other leukemia cell lines and in future preclinical pharmacodynamic and metabolomic studies on murine and xenografts models. The latter are demanded to increase the generalizability, translational potential, and overall impact of our findings.

The synergistic nature of the cytarabine-adavosertib drug pair in Jurkat was also established via densitometric analysis of γH2AX phosphorylation (Fig. 5D), a key player in DNA damage response (DDR) that repairs double strand breaks (DSBs). The combination led to a 7.9-fold change when compared to cytarabine, a 6.7-fold change when compared to adavosertib, and a 3.5-fold change when compared to the sum of both single agents. γH2AX is a biomarker of accumulated DNA damage leading to cell death or cell arrest. Our findings contrast previous studies [56] where an absence of WEE1 by siRNA decreased the speed of the replication fork and depletion of minichromosomal maintenance proteins (MCM4) from chromatin causing γH2AX to have a high expression. A qualitative analysis also suggested that adavosertib and cytarabine could alter phosphorylation γH2Ax and the cell cycle progression protein pCDC2 [18]. Our findings show that cytarabine (63 nM) increased phosphorylation of CDC2 (Fig. 5E) which causes cell cycle arrest to temporarily permit DNA repairs. In Jurkat, adavosertib exerted its inhibition of WEE1 leading to the expected decrease in pCDC2 seen (Fig. 5D). Although in different time frames, our results resemble a study using siRNA of WEE-1 with cytarabine on TF1 (Erythroleukemic cell line from blood) on CDC2 phosphorylation [17]. Since CDC2 can’t be dephosphorylated by constant exposure to the effects of the phosphorylation cascade caused by DNAdt we witness a high pCDC2 signal with cytarabine. In contrast, adavosertib and combination treatments allow the phosphorylated cascade to be interrupted by the inhibition of WEE1, permitting CDC25c to dephosphorylate CDC2 for cell cycle progression altogether leading to a mitotic catastrophe.

And as previously stated, the combinatorial synergistic efficacy of cytarabine-adavosertib in Jurkat and T cells may also be due to the relative expression or activation states of the WEE1 target proteins CDC2 and CDK1 [53].

Our Jurkat metabolome analysis revealed significant changes produced by the synergistic cytarabine-adavosertib combination in 22 metabolites, primarily amino acids interacting with the Krebs Cycle (Fig. 5B and D). The metabolites matched to 44 metabolic pathways with the top 25 enriched shown in Fig. 5C. Their relationship to amino acids metabolism and other cellular processes are diverse (Table 3). These interactions can cause cancer cells to utilize lipids and proteins to sustain their pathways. Other interacting pathways are responsible for cancer cell proliferation and progression, such as pyruvate metabolism. In a relevant study WEE1 inhibition or depletion impaired aerobic glycolysis and reduced cell viability in a dose-dependent manner in seven T-ALL cell lines, while sensitizing the cells to glutaminolysis inhibition [57]. Our results are also consistent with a previous metabolomic study where IC50 doses of adavosertib (MK1775) elicited in HPB-ALL cells global metabolic changes of crucial metabolic intermediates or products involved in aerobic glycolysis with the Warburg effect being the top process affected as per enrichment analysis [57]. Metabolites and processes unveiled by enrichment analysis in our study overlap with those conducted in HPB-ALL cells. Although, use of the ~IC50 of adavosertib led to decreases in HPB-ALL metabolites, our study in Jurkat using an ~IC25 concentration of adavosertib alone did not elicit significant changes in metabolites, yet in combination led to a consistent and significant decrease in the 22 metabolites when compared to the individual drug treatments (Fig. S2).

Analysis of the metabolites whose levels were statistically significantly reduced by the cytarabine-adavosertib combination (Fig. S2 and Fig. 6C) when compared to the control group yields specific insights into their roles in cancer, relation to main enriched processes (Krebs cycle, mitochondrial electron transport chain and gluconeogenesis) and the Warburg effect (Fig. 6C and Table 1). The analysis revealed that the synergistic drug combination elicited statistically significant decreases when compared to the control group in thirteen (13) (asparagine, b-alanine, glycine, valine, phenylalanine, glutaric acid, isoleucine, succinic acid, fumaric acid, malic acid. N-acetyl-aspartic acid, N-acetyl-l-glutamic acid and thapsic acid) of the twenty-two (22) metabolites (Fig. S2). Due to their relation to the Krebs cycle, the observed decreases in nine (9) (asparagine, glycine, valine, phenylalanine, isoleucine, succinic acid, fumaric acid and malic acid) of these thirteen (13) metabolites will reduce Krebs cycle activity and the concomitant generation of the reducing equivalents (NADH, FADH) required for the mitochondrial electron transport chain generation of ATP via oxidative phosphorylation [58]. Although decreased Krebs cycle and electron transport activity (two of the main enriched processes in Fig. 6C) may contribute to the metabolic reprogramming and mitochondrial respiratory injury as part of the Warbug effect [59-62] these can also lead to apoptosis, ferroptosis and cell death, adding another dimension to the combinatorial synergistic action of cytarabine-adavosertib [63,64]. In addition, decreased Krebs cycle activity can also lead to decreased accummulation of lactate via diminished conversion of oxaloacetate→phosphoenolpyruvate→pyruvate→lactate counteracting lactic acidosis and the lactate stimulation of cancer cell proliferation and suppression of anti-tumor immunity other hallmarks of the Warburg effect [59-62].

Furthermore, nine (9) of the latter metabolites (asparagine, glycine, valine, phenylalanine, isoleucine, succinic acid, fumaric acid, N-acetylaspartic acid and N-acetyl-glutamic acid) have been shown to promote tumor growth and/or metastasis in various types of cancer (non-small cell lung cancer, breast cancer, multiple myeloma, melanoma, pancreatic cancer, colorectal cancer, lymphoma, ovarian and brain tumors) and leukemia [32-45]. While dual roles have been reported for isoleucine and succinic acid [46-48]. In the case of asparagine, it promotes tumor progression and growth in non-small-cell lung cancer, ALL [32] and metastasis in breast cancer [33]. In solid tumors and breast cancer asparagine can support tumor cell survival when exogenous glutamine is depleted [32]. In Leukemia elevated asparagine has been found to be positively correlated with poor prognosis for this reason some treatments focus on asparaginase activation [38]. In turn, glycine deprivation inhibits proliferation in multiple myeloma [39]. Glycine along with proline are among the main amino acids used to form collagen [65] a major component of the tumor microenvironment and cancer fibrosis [66]. In leukemia glycine produces a favorable environment for blood cell and leukemic cell growth and expansion [40].

Valine catabolism is known to be essential for cancer cells as seen in colorectal cancer [41]. Being a branched amino acid valine and isoleucine are essential amino acid with vital roles in protein synthesis, energy production, membrane integrity and indirectly for nucleotide biosynthesis via the glutamate-glutamine axis necessary for tumor development and progression. Both were found in elevated concentrations in Kras-driven pancreatic tumors but not in other types of tumors [42]. A study in leukemia demonstrated that valine deficiency promotes apoptosis of leukemic cells [43] and promotes cancer complex formation [44]. Meanwhile, isoleucine differs from valine has an apparent anti-cancer function since at high concentrations it suppress proliferation of lung cancer [46].

The aromatic amino acid phenylalanine was seen to substitute tryptophan when depleted in melanoma cells for the biosynthesis of proteins [45]. While dual actions have been reported for succinic acid since it enhances cancer cell migration and promotes cancer metastasis [34], and displayed anti-cancer effects by inducing apoptosis in renal cancer [47]. In leukemia succinic acid had anti-proliferative activity and in vitro was seen to promote apoptosis in some leukemia cells [48]. In the case of fumaric acid in leukemia has been labeled as an oncometabolite and has cytoprotective effects in leukemia [35]. N-acetyl-aspartic acid (NAA) was shown to promote tumor growth and elevated concentrations of N-acetyl-aspartic acid and its enzyme, aspartate N-acetyl-transferase in patients with ovarian cancer and melanoma had worse overall survival than those with lower concentration [36]. Similarly N-Acetyl-L-glutamic acid (NAG) promotes tumor growth in lymphoma, ovarian and brain tumors [37].

In contrast to these 9 metabolites which overall had cancer promoting actions, two metabolites, B-alanine and malic acid, have been associated with anti-cancer roles B-Alanine was shown to suppresses breast cancer and increases sensitivity to doxorubicin [67], and malic acid and its derivatives could be used as anticancer agents for specific tumor treatments since in glioblastoma malic acid had a genotoxic activity [68]. No cancer related findings can be found for glutaric acid while a study of thapsic acid demonstrated its potential role as biomarker for early detection of colorectal cancer [69]. While in the case of glutamine, even though it has been documented that cancer cells are “addicted” given its multiple pro-cancer roles [52], its metabolism plays a central role in regulating uncontrolled tumor growth by modulating bioenergetic and redox homeostasis and serving as a precursor for the synthesis of biomass [70], and cancer cells rely on glutamine to interact with M1 and M2 macrophages to alter pro-inflammatory and anti-inflammatory responses which effects endothelial cells permitting tumor metastasis [71] its levels were decreased but not significantly by adavosertib, nor the cytarabine-adavosertib combination when compared to the control group in Jurkat (Fig. S2). Additional experiments and/or other synergistic cytarabine-adavosertib concentrations may unveil the significance of glutamine in the synergistc assessment in Jurkat. Most importantly, we can conclude that the observed decrease in the metabolites’ levels with pro-cancer roles produced by the adavosertib-cytarabine combination supports their proposed role promoting cancer, whilst pinpointing to additional mechanisms or targets for the antitumor efficacy of our synergistic drug combination.

In addition, five (5) of the thirteen (13) metabolites are glucogenic amino acids (asparagine, b-alanine, glycine, valine and phenylalanine) [72] an observation consistent with gluconeogenesis being one the three main enriched processes (Fig. 6C) affected by the drug combination. Indeed, six (6) of the 9 metabolites whose levels were decreased compared to control but not significantly are also glucogenic [72], a decrease expected to diminish gluconeogenesis. Although decreased gluconeogenesis may favor glycolysis it will also diminish the available intracellular glucose for the aerobic glycolysis of the Warburg effect in these leukemic cancer cells. A diminished Warburg effect may also serve as a complementary anticancer mechanism for the cytarabine-adavosertib synergistic drug combination. These metabolomic-based set of actions add further value to the claim by Garcia et al. (2018) [53] that the combinatorial chemosensitizing efficacy of the cytarabine-adavosertib drug combination in leukemic Jurkat cells encompasses more than their combined DNA damage and G2/M arrest inhibition. Hence the pharmacodynamic and metabolomics findings of this study can be shedding light on the need to consider all the following mechanisms in explaining the combinatorial synergistic efficacy of cytarabine-adavosertib and other drug pairs: (1) combined DNA damage and G2/M arrest inhibition, G1/S arrest inhibition leading to mitotic catastrophe, (2) diminished Krebs cycle activity with decreased reducing equivalents for ATP synthesis, diminished electron transport chain and oxidative phosphorylation ATP synthesis leading to apoptosis and/or ferroptosis; and, (3) diminished lactate accumulation and decreased gluconeogenesis leading to decreased intracellular glucose for the aerobic glycolysis of the Warburg effect. Future studies must address novel mechanisms involved in the Warburg effect and the relationship to synergistic drug actions, such as the role of hypoxia-inducible factor (HIF-1), microRNAs, regulation of mitochondrial pyruvate uptake via MCT4, and the regulation of facilitated cellular glucose uptake via GLUT1 [60-62,73].

We can also mention from the metabolomics data in Jurkat, that the adavosertib-cytarabine synergistic combination treatment also reduced nucleotide metabolisms necessary for DNA replication, RNA synthesis, cellular energy, and interactions. Increased nucleotide metabolism leads to uncontrolled growth of tumors [74,75]. Glutathione metabolism is important for cell differentiation, proliferation, apoptosis, and progression of many diseases. As stated before ancer cells modify glutamine and glutamate metabolism to maintain cell growth and proliferation, while arginine biosynthesis has a key role in cell survival and proliferation in normal and malignant cells [75]. In turn, fumaric acid promotes tumor growth through diverse signaling functions and succinic acid accumulation can impact gene expression regulation and promote tumorigenesis. In addition, high acetyl-CoA shifts cells to a pro-anabolic state increasing expression of genes for cell growth and proliferation, including glycolytic enzymes. Acetyl-CoA can be generated from the oxidation of pyruvate, fatty acid, oxidation degradation of leucine, isoleucine, and tryptophan, or mitochondrial enzyme aceyl-CoA synthetase short-chain family, member 1 (ACSS1)-mediated conversion of acetate. [75,76]. Combination treatment decreased leucine and isoleucine, and threonine which via a dehydrogenase pathway is degraded to acetyl-CoA. Additional pathways relevant to cancer include metabolic recycling of ammonia via glutamate dehydrogenase which supports biomass in breast cancers [77], porphyrin accumulation:, [78] ketone body metabolism, [79] gluconeogenesis, [80] and Warburg metabolism [81].

## 5. Conclusion

Our study provides a comprehensive analysis of the synergistic interaction between cytarabine and adavosertib in the CCRF-CEM and Jurkat ALL model systems. The approach provides an important framework for future synergism analysis in high throughput in vitro drug screening assays. The analysis permitted expansion and identification of the range of potential useful cytarabine-adavosertib synergistic combinations for pre-clinical and future clinical translation. Identification and prioritization of a high degree of synergy cytarabine-adavosertib combination allowed assessment of effects at cellular, molecular and metabolome levels. The effects on two endpoints demonstrate cancer cell selective synergy, an added value to the decreased dosing, side effects and tolerance advantages of synergistic drug combinations. In summary, we conclude that the high synergistic efficacy combination of cytarabine (63 nM) and adavosertib (97 nM) was associated with remarkable alterations in metabolites related to the Krebs cycle in Jurkat. The metabolic pathways and processes are related to gluconeogenesis, amino acids, nucleotides, glutathione, electron transport and Warburg effect. All above relate to cell survival, apoptosis, and cancer progression. Our findings could pave the way for novel biomarkers in treatment, diagnosis, and prognosis of leukemia and other cancers.

## Supplementary Material

1

2

3

## Figures and Tables

**Table 1:** Summary of IC_50_ ( ± SEM) values and Fold-shifts in Concentration Inhibition Curves of Adavosertib and Cytarabine, Alone and in Combination in the CCRF-CEM and Jurkat Cell Lines.

CCRF-CEM Cell Line			
Adavosertib IC_50_ ± SEM (nM)	Cytarabine (nM)	Combination Fold Reduction in IC_50_	N
51 ± 4	24	2.02	8
70 ± 1	13	1.37	8
86 ± 4	7	1.20	8
97 ± 4	4	1.06	8
102 ± 2	2	1.01	8
102 ± 2	1	1.01	8
103 ± 5	0	————	8
Cytarabine IC_50_ SEM (nM)	Adavosertib (nM)	Combination Fold Reduction in IC_50_	N
11 ± 1	75	8.18	8
28 ± 2	48	3.21	8
48 ± 2	30	1.88	8
90 ± 5	0	————	8
Jurkats			
Adavosertib IC_50_ ± SEM (nM)	Cytarabine (nM)	Combination Fold Reduction in IC_50_	N
94.7 ± 26	31	3.91	8
164.1 ± 11	16	2.26	8
239 ± 5	8	1.55	8
295.9 ± 8	4	1.25	8
333.6 ± 19	2	1.11	8
370.4 ± 13	0	————	8
Cytarabine IC_50_ ± SEM (nM)	Adavosertib (nM)	Combination Fold Reduction in IC_50_	N
16.2 ± 1	162	9.86	8
30 ± 2	97	5.32	8
52.6 ± 3	58	3.04	8
159.7 ± 8	0	————	8

**Table 2:** Summary of CCRF-CEM and Jurkat Top 15 Synergy Scores per Model via Combenefit and SynergyFinder Plus. Cytarabine and adavosertib concentrations are shown in the first two rows of each leukemic cell line. The rows indicate the different synergy scores per synergy model and the mean synergy score is shown in the bottom row. The highest mean synergy score across all models is shown in bold and corresponds to 63 nM cytarabine with 97 nM adavosertib in Jurkat.

CCRF-CEM															
**cytarabine (nM)**	84	84	84	84	45	**45**	45	45	24	**24**	24	13	13	7	7
**adavosertib (nM)**	30	48	75	119	30	**48**	75	119	48	**75**	119	75	119	75	119
Models	Combenefit Synergy Score (%)														
**Loewe**	14	17	12	6	4	**21**	20	10	2	21	12	7	12	−17	8
**Bliss**	32	36	25	10	27	**48**	40	17	22	38	18	18	16	−11	10
**HSA**	34	45	48	21	31	**62**	50	21	26	40	19	18	17	−11	10
**Mean Score**	26.7	32.7	28.3	12.3	20.7	**43.7**	36.7	16	16.7	33	16.3	14.3	15	−13	9.3
Models	SynergyFinder Plus Synergy Score (%)														
**Loewe**	16.3	17.3	10.4	3.2	17.8	30.2	23.1	7.1	17	**36.4**	10.8	30.3	11.9	10.1	10.3
**Bliss**	33.5	42.9	42.9	13.2	27.8	54.1	66	21.2	22.8	**65.5**	22	45.8	19.08	15.8	13.8
**HSA**	38.7	50.1	53.1	24.9	35.4	63.4	76.4	24.8	28.4	**70.8**	23.6	49.6	20.3	29.8	15.3
**ZIP**	36.6	46	42.2	13.4	28.9	60.2	64.7	21.1	26.6	**64.5**	22.5	44.5	20.4	15.5	14.1
**Mean Score**	31.3	39.1	37.2	13.7	27.5	52	57.6	18.6	23.7	**59.3**	19.7	42.6	17.9	17.8	13.4
Jurkat															
**cytarabine (nM)**	63	125	31	**63**	125	16	31	63	125	4	8	16	31	63	125
**adavosertib (nM)**	58	58	97	**97**	97	162	162	162	162	270	270	270	270	270	270
Models	Combenefit Synergy Score (%)														
**Loewe**	61	59	50	**85**	48	46	84	76	30	24	56	73	64	36	12
**Bliss**	63	75	50	**91**	78	47	88	95	79	26	61	83	89	88	73
**HSA**	63	75	50	**91**	78	47	88	95	79	26	61	83	89	89	79
**Mean Score**	62.3	69.7	50.0	**89.0**	68.0	46.7	86.7	88.7	62.7	25.3	59.3	79.7	80.7	71.0	54.7
Models	SynergyFinder Plus Synergy Score (%)														
**ZIP**	64.9	74.9	53.7	**90.4**	78.3	48.2	89.3	94.6	79.3	28.6	60.6	82.5	87.8	88.1	73.8
**Loewe**	62.5	58.9	52.6	**84.7**	45.7	46.7	84.7	54	22.2	26.7	55	73.1	32.1	14.1	8.4
**Bliss**	65.2	74.9	53.6	**90.7**	78.3	48.2	88.6	95.6	79.3	28.8	59.5	83.1	88.5	88.5	73.8
**HSA**	65.1	74.9	53.6	**90.6**	78.3	48.2	88.6	95.5	79.3	28.8	59.5	83.1	88.5	88.7	79.8
**Mean Score**	64.4	70.9	53.4	**89.1**	70.2	47.8	87.8	84.9	65	28.2	58.6	80.5	74.2	69.9	58.9

**Table 3:** Principle amino acids affected by treatments in their respective metabolism and related processes where they interact in the biological functions of the cell.

Amino Acids	Amino Acids Metabolism	Related Processes
**Beta-alanine**	Beta-Alanine; Histidine; and Aspartate Metabolisms	Pyrimidine Metabolism; Propanoate Metabolism
**Fumaric acid**	Aspartate; Arginine; Proline; Phenylalanine and Tyrosine Metabolisms.	Urea Cycle; Warburg Effect; Purine Metabolism; Citric Acid Cycle; Mitochondrial Electron Transport Chain and Pyruvate metabolism.
**Pyroglutamic acid**	Glutathione Metabolism	N/A
**Succinic acid**	Glutamate; Arginine and Proline Metabolism. Valine, Leucine and Isoleucine Degradation.	Warburg Effect; Oxidation of Branched Chain Fatty Acids; Ketone Body Metabolism; Butyrate Metabolism; Phytanic Acid Peroxisomal Oxidation; Carnitine Synthesis; Citric Acid Cycle; Mitochondrial Electron Transport Chain and Pyruvate metabolism.
**Glycine**	Glutamate; Glycine; Serine; Glutathione; Alanine; Methionine; Arginine and Proline Metabolisms	Ammonia Recycling; Purine Metabolism; Carnitine Synthesis; Porphyrin Metabolism and Bile Acid Biosynthesis.
**Isoleucine**	Valine, Leucine and Isoleucine Degradation.	N/A
**Alanine**	Selenoamino Acid; Glutamate; Tryptophan; Glycine; Serine; Glutathione and Alanine Metabolisms.	Glucose-Alanine Cycle; Urea Cycle and Pyruvate metabolism.
**Asparagine**	Aspartate Metabolism	Ammonia Recycling
**Glutamic Acid**	Glutamate; Tryptophan; Glycine; Serine; Glutathione; Alanine; Cysteine; Aspartate; Arginine; Proline; Aspartate; Phenylalanine; Tyrosine; Beta-Alanine; Histidine Metabolisms and Lysine, Valine, Leucine and Isoleucine Degradation.	Amino Sugar Metabolism; Nicotinate and Nicotinamide Metabolism; Glucose-Alanine Cycle; Urea Cycle; Ammonia Recycling; Warburg Effect; Purine Metabolism; Folate Metabolism; Arachidonic Acid Metabolism; Propanoate Metabolism; Malate-Aspartate Shuttle.
**Glutamine**	Glutamate; Phenylacetate and Aspartate Metabolism.	Amino Sugar Metabolism; Nicotinate and Nicotinamide Metabolism; Urea Cycle; Ammonia Recycling; Warburg Effect; Purine Metabolism and Pyrimidine Metabolism.
**Leucine**	Valine, Leucine and Isoleucine Degradation	
**Methionine**	Methionine; Glycine and Serine Metabolisms	Betaine Metabolism; Spermidine and Spermine Biosynthesis
**Phenylalanine**	Phenylalanine and Tyrosine Metabolisms	
**Proline**	Arginine and Proline Metabolisms	
**Threonine**	Glycine and Serine Metabolisms and Threonine Degradation.	2-Oxobutanoate Degradation
**Tyrosine**	Phenylalanine and Tyrosine Metabolisms	Catecholamine Biosynthesis and Thyroid hormone synthesis.
**Valine**	Valine, Leucine and Isoleucine Degradation	Propanoate Metabolism
**Malic Acid**	N/A	Malate-Aspartate Shuttle and Gluconeogenesis
**N-acetyl aspartic acid**	Aspartate Metabolism	N/A

## Data Availability

Data will be made available on request. For original CIC and metabolome data, please contact hmaldonado1@gmail.com, or gaby92984@gmail.com. The raw metabolomic data is available in metabolomicsworkbench.org.
