# Supplementary material for: Synergistic interactions of cytarabine-adavosertib in leukemic cell lines proliferation and metabolomic endpoints
Source: Biomed Pharmacother. Author manuscript; Available in PMC 2023 Oct 1. (PMC10530627; doi:10.1016/j.biopha.2023.115352)
Supplement: 1 [file NIHMS1931056-supplement-1.docx]

**Supplementary Figure Legends**

**Figure S1.  Original representative Western blot of the effects 63nM cytarabine, 97nM adavosertib and their combination before and after 24hrs exposure of Jurkat cells (See Figure 5).** Panel A shows the membrane India ink staining. Panel B shows full phosphorylation and protein expression of γH2AX, pCDC2 and tubulin after processing as described in the Materials Methods section. Each lane was loaded with 10µg of protein:  Lane 1, 0hrs control; Lane 2, 24hrs Control; Lane 3, 63nM cytarabine; Lane 4, 97nM adavosertib; and, Lane 5, Combination of 63nM cytarabine-97nMadavosertib.

**Figure S2. Statistical Analysis of the 22 Amino Acid Metabolites affected by the drugs alone and in the combination.** This figure reveals the statistical analysis of the effects on amino acid metabolite levels exerted by cytarabine (63nM), adavosertib (97nM) and their combination treatment compared to control group. Statistical analysis was conducted using Tukey’s test with the standard error mean (SEM) (n=5). Statistical significance: *, p<0.05; **, p<0.01; ***, p<0.001; ****, p<0.0001.
