# Supplementary figures and images for: Synergistic interactions of cytarabine-adavosertib in leukemic cell lines proliferation and metabolomic endpoints
Source: Biomed Pharmacother. Author manuscript; Available in PMC 2023 Oct 1. (PMC10530627; doi:10.1016/j.biopha.2023.115352)

**A**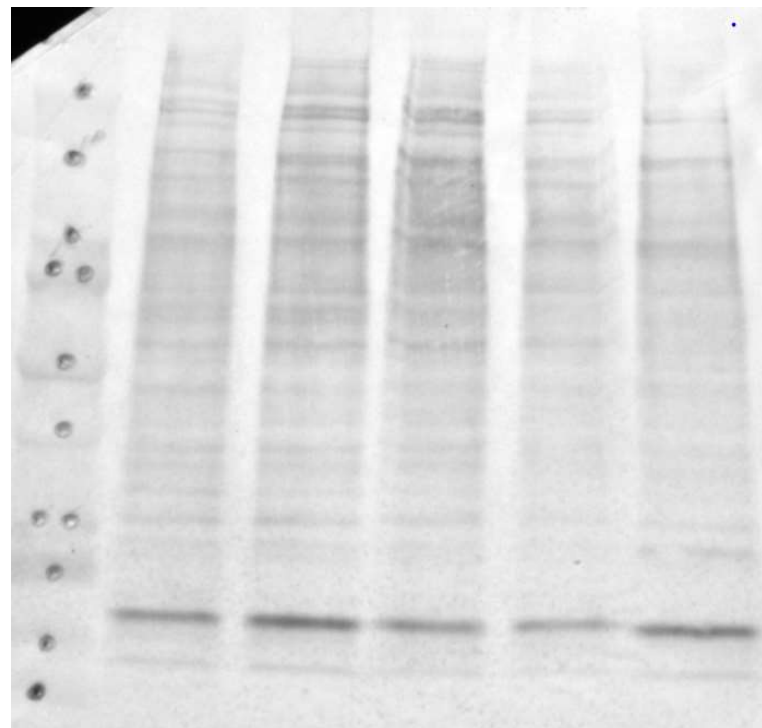

MW  
markers

1 2 3 4 5

**B**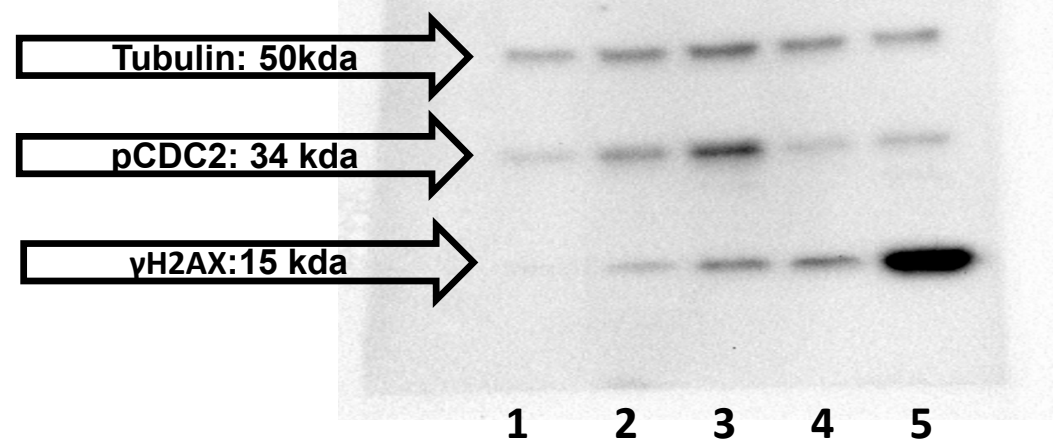

1 2 3 4 5

Supplement: 2 [file NIHMS1931056-supplement-2.pdf]

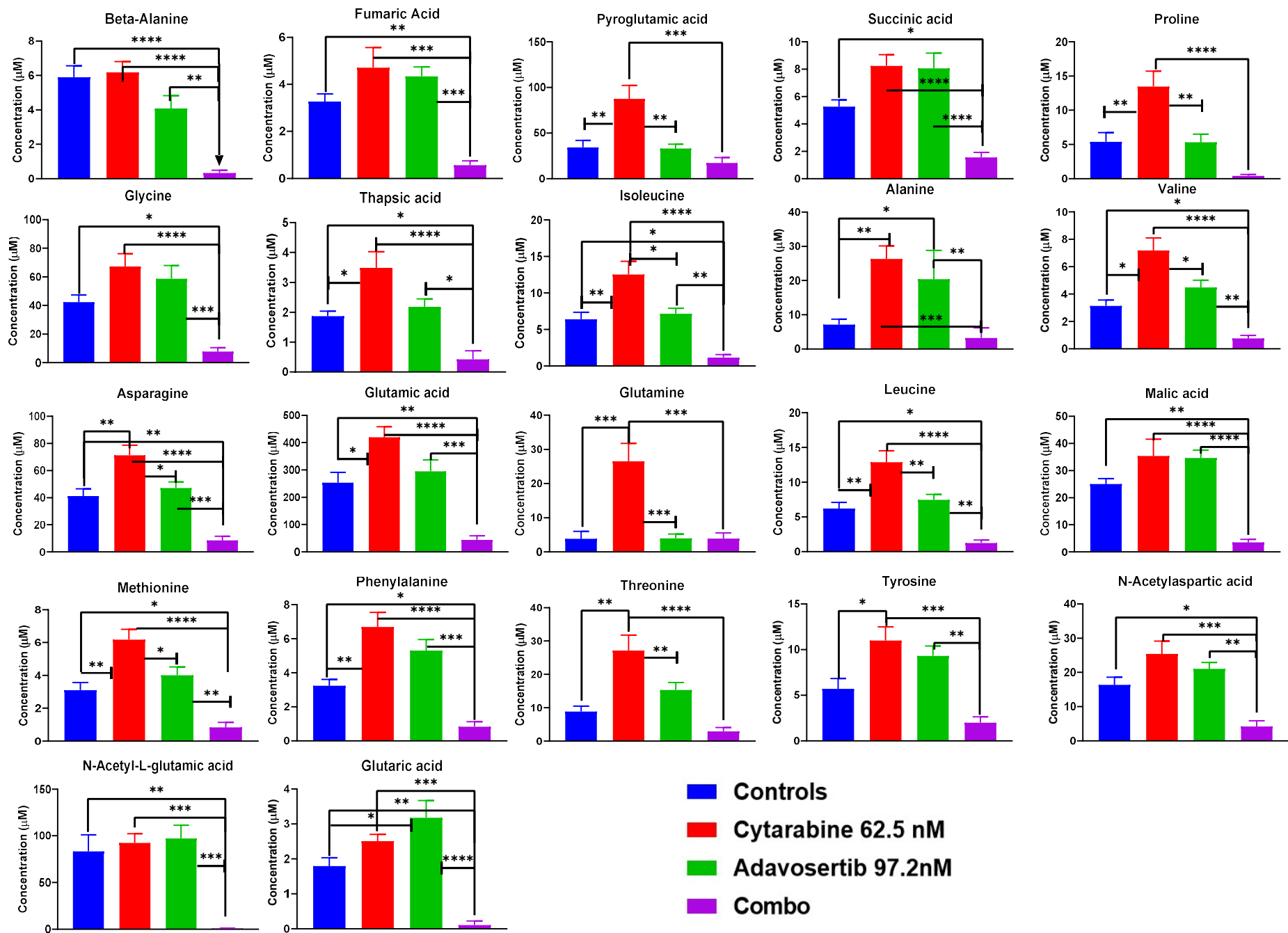

Supplement: 3 [file NIHMS1931056-supplement-3.pdf]
